# Classification of breed combinations for slaughter pigs based on genotypes—modeling DNA samples of crossbreeds as fuzzy sets from purebred founders

**DOI:** 10.3389/fgene.2023.1289130

**Published:** 2023-12-04

**Authors:** H. Vinje, H. K. Brustad, A. Heggli, C. A. Sevillano, M. Van Son, L. E. Gangsei

**Affiliations:** ^1^ Faculty of Chemistry, Biotechnology and Food Science, Norwegian University of Life Sciences, Ås, Norway; ^2^ Oslo Center of Biostatistics and Epidemiology, Oslo University Hospital, Oslo, Norway; ^3^ Animalia AS, Oslo, Norway; ^4^ Topigs Norsvin Research Center, Beuningen, Netherlands; ^5^ Norsvin SA, Hamar, Norway

**Keywords:** slaughter pigs, breed classification, crossbreeds, fuzzy classification, single-nucleotide polymorphism, partial least squares, quadratic discriminant analysis, ADMIXTURE

## Abstract

In pig production, the production animals are generally three- or four-way crossbreeds. Reliable information regarding the breed of origin of slaughtered pigs is useful, even a prerequisite, for a number of purposes, e.g., evaluating potential breed effects on carcass grading. Genetic data from slaughtered pigs can easily be extracted and used for crossbreed classification. In the current study, four classification methods, namely, random forest (RF), ADMIXTURE, partial least squares regression (PLSR), and partial least squares together with quadratic discriminant analysis (PLS-QDA) were evaluated on simulated (*n* = 7,500) genomic data of crossbreeds. The derivation of the theory behind PLS-QDA is a major part of the current study, whereas RF and ADMIXTURE are known and well-described in the literature. Classification success (CS) rate, square loss (SL), and Kullback–Leibler (KL) divergence loss for the simulated data were used to compare methods. Overall, PLS-QDA performed best with 99%/0.0018/0.002 (CS/SL/KL) vs. 97%/0.0084/0.051, 97%/0.0087/0.0623, and 17%/0.068/0.39 for PLSR, ADMIXTURE, and RF, respectively. PLS-QDA and ADMIXTURE, as the most relevant methods, were used on a real dataset (*n* = 1,013) from Norway where the two largest classes contained 532 and 192 (PLS-QDA), and 531 and 193 (ADMIXTURE) individuals, respectively. These two classes were expected to be dominating *a priori*. The Bayesian nature of PLS-QDA enables inclusion of desirable features such as a separate class “unknown breed combination” and informative priors for crossbreeds, making this a preferable method for the classification of breed combination in the industry.

## 1 Introduction

Several meat production livestock systems rely on crossbred animals. In pig production, the production animals are generally three- or four-way crossbreeds (CBs). There are several reasons for using CB in meat production, in particular to benefit from heterosis and breed complementarity and to be flexible in creating different products for different markets (Smith, 1964; Dickerson, 1973; Sellier, 1976). In contrast to meat production from other domestic animals, such as cattle, breed information for production pigs is not logged through the production chain, and hence, no, or at least incomplete, controls exist for the breed combination of individual slaughtered pigs.

Verification of a true CB combination is beneficial at different steps of the pig production chain. For instance, having control of CB breed origin will allow for the inclusion of CB performance from different sources after verification in the selection program of a pig breeding company. Including CB performance is desirable for achieving a sizable genetic progress for traits showing a genetic correlation between PB performance and CB performance that is lower than unity (Wientjes and Calus, 2017). Breed and crossbreeding also have a significant effect on meat quality traits (Kim et al., 2020) and the distribution of undesired mutations like halothane and Rendement Napole genes (Hamilton et al., 2000). For these reasons, verification of the breed of origin will be valuable for abattoirs and retailers. Finally, it is natural to assume that breed origin might be an unobserved nuisance factor for carcass grading; see Gangsei et al. (2018) for an elaborating discussion. If CB combinations were known for dissected carcasses, it would facilitate the evaluation of potential biases regarding the grading of different breed combinations.

The problem addressed in the current study is the classification of breed combination for individuals in the CB population based on genetic data, i.e., single-nucleotide polymorphisms (SNPs) from a 50-K SNP chip. Such genomic data have become cheaper and more accessible due to rapid developments in technology, and the number of application areas has exploded within different parts of the natural sciences, including ancestry classification tasks.

Most methods used to infer breed combinations in pig populations with genomic data were originally developed for inferring human ancestry. ADMIXTURE (Alexander et al., 2009) is one of the most popular methods used to classify individuals with an unknown ancestry into discrete populations and was developed for human populations but has been used extensively in pigs to trace commercial (Huang et al., 2014) or indigenous pig breeds (Mujibi et al., 2018; Dadousis et al., 2022; Kim et al., 2022; Yin et al., 2023). Principal component-based algorithms for determining the ancestry have also been developed, such as PCAdmix (Brisbin et al., 2012), and applied in pig populations (Schleimer et al., 2022). As pig breeding is far from human genetics, new methods are needed for better classification. An approach has been developed to assign alleles in three-way CB pigs to their PB of origin; the approach has high accuracy, but as it infers local ancestry, it is highly computationally demanding (Sevillano et al., 2016; Vandenplas et al., 2016). Another study tried using random forest for this purpose in pigs; however, the method did not accurately estimate breed composition for the breeds in question with the available markers (Chinchilla-Vargas et al., 2021). Recently, an interesting study was published showing the advantage of partial least squares regression (PLSR) and partial least square–discriminant analysis (PLS-DA) for global ancestry identification of pig breeds (Miao et al., 2023). The results showed that a wide range of breeds can be discriminated using these methods and that alternatives to human-developed methods can be beneficial for the pig industry. However, the study was restricted to the classification of PBs and treated CBs as similar to unknown breeds.

When assuming that grandparents are PB animals from a set of known PBs with known origin, i.e., breed, there will always be a possibility that CB individuals might have one or more grandparents from breeds outside the set with predefined known breeds. Ideally, classification methods should be able to identify such individuals and classify them as an “unknown breed combination.” All the aforementioned methods, except PLS-DA, lack the ability to incorporate such a feature, and further elaboration is needed to achieve this objective.

The novelty of the present study is to derive the theoretical basis for partial least squares with quadratic discriminant analysis (PLS-QDA) (Boulesteix, 2004; Hastie et al., 2009) used for CB classification based on the following steps: i) PLS was used as a replacement for PCA/MDS as the primary dimension reduction method for SNP data, ii) additional variance was incorporated by modeling the proportion of DNA inherited from each grandparent as a random variable, and iii) the Bayesian nature of QDA was utilized to incorporate informative priors for CB classes and the possibility to include a class “unknown breed origin”. The variance in proportion of inherited DNA was combined with breed-specific variances for PBs in order to achieve CB-specific covariance matrices for PLS components. CB-specific covariance matrices enable the use of QDA as a replacement for the more common linear discriminant analysis (LDA). An additional asset facilitated by PLS-QDA is visualization of the behavior of decision boundaries in a low (<3) dimensional space.

The overall aim of this study is to evaluate the crossbreed classification of commercial finisher pigs based on genomic data from a 50-K (Illumina) SNP chip. Two other well-known classification methods, random forest (RF) and ADMIXTURE, were compared with PLSR and PLS-QDA.

## 2 Materials and methods

### 2.1 Materials

#### 2.1.1 Genomic data

The genotypes used in this study are data collected from the pig breeding companies Norsvin (Norway) and Topigs Norsvin (the Netherlands). Animals were genotyped using a custom GeneSeek 50-K (Illumina) SNP chip (Lincoln, NE, Unites States). Of these, 23,070 SNPs are used routinely by Topigs Norsvin and constitute the raw SNP data in the current study. Based on PB animals (*n* = 4,014), it was observed that from five different PBs (see details in the following section), the minimum call rate was 0.997 and minor allele frequency (MAF) was 0.045, well inside the limits used by Tusell et al. (2020) at 0.9 for call rate and 0.01 for MAF.

For each SNP, the most frequent allele in 4,014 PBs was identified. SNPs were coded into numeric vectors with zero for the homozygous genotype of the most frequent allele at the SNP in question, one for the heterozygote, and two for the homozygous genotype of the least frequent allele.

Data from five PBs (*n* = 4,014) are used as training data for all models. They also constitute the basis for data simulation; see the paragraphs in the following section for details. PBs are Landrace (abbreviation “L,” *n* = 1,000), Large White (“W,” n = 1,000), Duroc (“D,” *n* = 1,000), and Hampshire (“H,” *n* = 14), which are dominating PBs in the Norwegian pig population. For the exploration of uncertainty measures and generalizations of the methods, a fifth breed, Pietrain (“P,” *n* = 1,000), not present in Norway, was incorporated into the study. In the present study, the term “breed” is used extensively. In practice, the PBs described previously might be viewed as sub populations/lines primarily present in the Norwegian pig population. In addition to the SNP data from PB individuals, SNP data from 1,013 slaughter pigs with unknown breed origin were used to examine model behavior.

#### 2.1.2 Breeds and breed combinations

The focus of this study is to classify the breed combinations of founders (F0 generation) observed in the commercial finisher pigs (F2 generation) based on genotypes from the F2 generation. It is assumed that all F0 individuals are PB.

When *q* PBs are present, there are *q* ^4^ (625 for *q* = 5 and 256 for *q* = 4) different unique breed permutations in the F2 generation. For example, *“LWDD”* indicates L and W as the grandfather and grandmother of the maternal line, respectively, and D as the grandfather and grandmother at the paternal line, which is the most common Norwegian finisher breed combination. The pure breeds Duroc and Hampshire are typically the paternal line for production pigs.

The *q* ^4^ unique permutations constitute a total of $n_{\mathrm{comb}}=\left(\begin{array}{c} q+4-1\\ 4 \end{array}\right)$ (70 for *q* = 5 and 35 for *q* = 4) unique breed combinations when the sequence of grandparents is not taken into account. Combinations are given with letters in descending alphabetical order. For example, the combination *“DDLL”* contains the permutations {*“LLDD*,*” “LDLD*,*” “LDDL*,*” “DDLL*,*” “DLLD*,*” “DLDL”*}, etc.

### 2.2 Simulation of SNP data for crossbreeds

In order to test the accuracy of classification methods, CB data with a known breed origin form a prerequisite. For the current study, such data were nonexistent, and data simulation was used to obtain relevant test datasets for the methods.

SNP data from the PBs (*n* = 4,014) were used as the input for the simulation. The output was combinations of the SNP data in accordance with known CB combinations. One simulated test set, TestP−, originates from PBs omitting Pietrain. The other test set, TestP+, consists of breed combinations with at least one Pietrain grandparent. For each CB combination, we performed 100 simulations, resulting in 3,500 simulations for both test sets, TestP− and TestP+.

**FIGURE 1:**
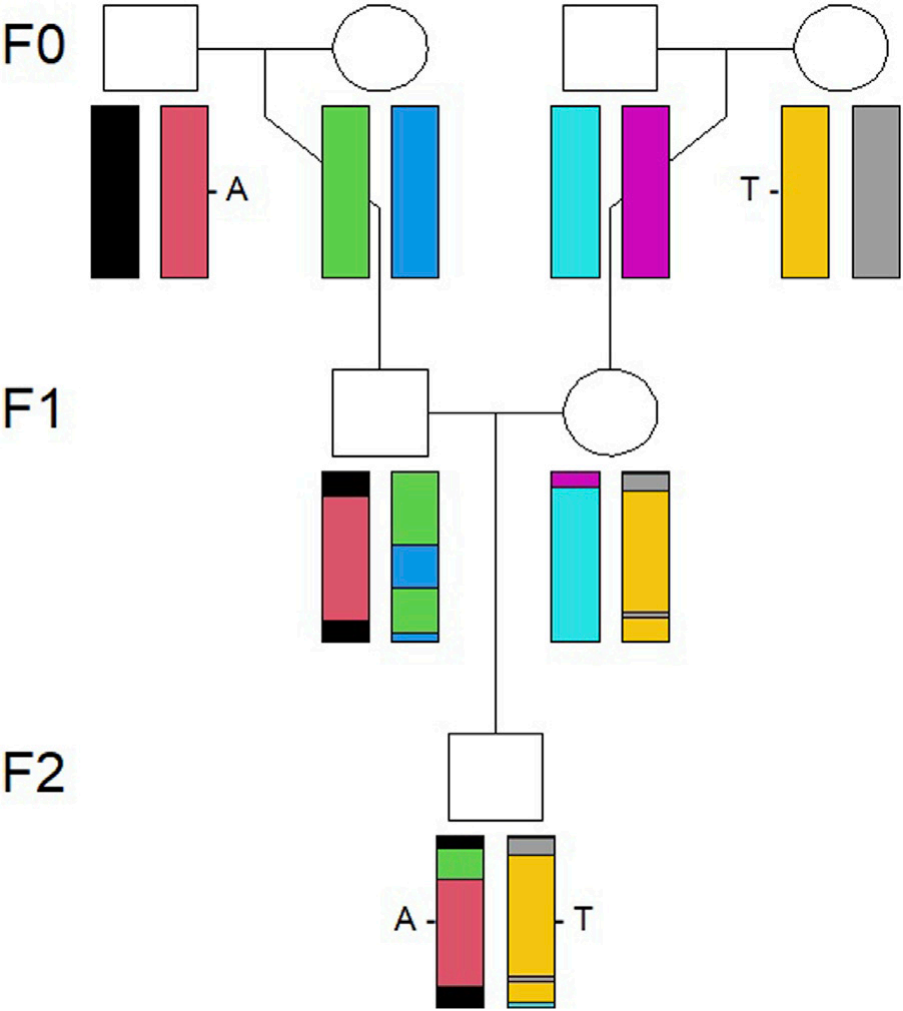
Illustration of how a chromosome pair is inherited IBD from the F0 to F2 generation. The genomes recombine from the F0 to F1 generation and then to the individual in the F2 generation, visualized by the combination of colors of the chromosomes.

Simulations were conducted using R packages and functions described in Vigeland (2021). The first step in the simulation procedure was to simulate an identical by descent (IBD) pattern along the genome for an individual in the F2 generation, as shown in Figure 1, based on a pedigree connecting the F0 and F2 generations. An IBD pattern shows how different parts of an individual’s genome are inherited through descent from previous generations, from grandparents in the present study, using information on how the chromosomes recombine. The recombination is a stochastic process along the genome. A prerequisite for simulation of IBD patterns is a recombination map which relates the cumulative genetic distance, in centimorgans, to the cumulative physical distance, in bases, along the genome. The genetic map provided by Tortereau et al. (2012) was used as the basis for the recombination map.

The second step was to sample SNP data for the F2 individual, conditional on the simulated IBD pattern and the CB combination from the individual in question. Thus, the F0 generation was randomly selected among the 4,014 PB individuals, and then, their allele data (nucleotide bases A, C, T, and G) were transferred to the F2 individual, in accordance with the simulated IBD pattern for each SNP. Consequently, the simulated data are different combinations of the original allele data from the 4,014 PB pigs.

### 2.3 Evaluation of classification results

The typical goal of classification is to assign an observation to one out of a distinct set of classes. A problem arises when the goal is to classify in between such distinct classes. We will regard the CB pigs as such fuzzy sets (Zadeh, 1965), i.e., the class of CB pigs is regarded as a continuum of grades of membership in the PB classes.

Four classification methods were tested: random forest (RF), ADMIXTURE, partial least squares regression (PLSR), and partial least squares with quadratic discriminant analysis (PLS-QDA); see following sections for details. Only PB individuals were used for training the four classification methods. We applied two different training datasets, one consisting of all PB individuals and another omitting the Pietrain data, referred to as TrainP+ and TrainP−, respectively.

For the two simulated test sets, TestP+ and TestP−, breed combinations were classified using all four aforementioned methods trained on the two training sets (TrainP− and TrainP+), leading to a total of 16 (“four methods” × “two training data sets” × “two test data sets”) combinations of methods, training, and test data. For each combination, 3,500 individuals were classified, i.e., 35 “CB combinations” × 100 “individuals per combination”. The real data from CBs with unknown breed origin (*n* = 1,013), denoted “TestR”, were classified using ADMIXTURE and the PLS-QDA method. The results were used to examine the effect of an informative prior distribution in the latter and whether the methods provided useful and credible results in a practical setting.

All breed combinations might be represented by a vector *Δ* of length *q* (q = 4 in TrainP− and q = 5 in TrainP+) whose elements are the proportions of grandparents from each of the *q* PBs, in alphabetic order, i.e., “D,” “H,” “L,” “P,” and “W.” For example, an individual with breed combination *DDLW* will have $\Delta =\frac{1}{4}{\left[20101\right]}^{t}$ . Consequently, the elements of *Δ* are quarters, which sum to 1. All four classification methods give predictions for *Δ* which denoted $\hat{\Delta }$ . Even if the elements of *Δ* are quarters, the predictions are proportions, i.e., $0\leq {\hat{\delta }}_{\mathrm{new}}^{j}\leq 1$ and $\sum_{j}^{q}\hat{\delta_{j}}=1$ , but the elements ( $\hat{\delta_{j}}$ ) are not necessarily in quarters.

Two statistics are calculated for the evaluation of different methods based on simulated data where the true breed combinations (*Δ*) are known. The square loss for a new prediction is given by $\sum_{j}^{q}{\left(\delta_{j}-\hat{\delta_{j}}\right)}^{2}$ , and the Kullback–Leibler divergence (Kullback and Leibler, 1951) is the divergence between the two multinomial distributions for 4*Δ* with probability vectors *Δ* and $\hat{\Delta }$ , respectively.

Hard classifications for RF, ADMIXTURE, and PLSR were achieved by choosing the breed combination with either the shortest Kullback–Leibler divergence or minimal square loss. For PLS-QDA, the hard prediction is the CB class with largest posterior probability.

### 2.4 Classification methods

#### 2.4.1 ADMIXTURE and random forest

ADMIXTURE (Alexander et al., 2009) is an algorithm and software tool for the maximum likelihood estimation of individual ancestries, usually used for humans but also possible to apply to other species like pigs. ADMIXTURE 1.3 software (avid H. Alexander et al., 2020) was used for this analysis in a supervised mode with K-values set to 4 (for TrainP−) and 5 (for TrainP+).

RF is a widely used classification method built on the theory of tree-structured classifiers. An RF consists of a collection of *K* tree-structured classifiers, where *K* is usually a large number. In the end, all trees vote for their preferred class and RF classifies to the class with most votes (Breiman, 2001; Hastie et al., 2009). The Hampshire data were oversampled in the tree-growing process, inversely proportional to their abundance compared to other breeds (1,000/14). RF analysis was conducted via the “randomForest” package (Liaw and Wiener, 2022) in R. Only training data were used for tuning hyper parameters, with the out-of-bag (OOB) error as the performance measurement. The major hyper parameters to tune are the number of drawn candidate variables in each split (*m*_*try*_), the number of observations drawn for each tree (sample size), node size, and number of trees (*K*) (Probst et al., 2019). We used $m_{\mathrm{try}}=151\approx \sqrt{23070}$ , number of trees *K* = 100, sample size 100, and node size 1, which gave OOB errors equal to 0 for both TrainP+ and TrainP−.

#### 2.4.2 Partial least squares regression

Partial least squares (PLS) is a supervised method where breed information is taken into account. Wold et al. (2001) offers an overview over the fundamental principles of PLS. The basic idea of PLS regression (PLSR) is to find the multidimensional directions in the predictor variable space, i.e., the SNP (**X**) that explains the maximum multidimensional variance direction in the response, i.e., the breed (**Y**).

We apply a multivariate response matrix **Y** (*n* × *q*) for the PLS regression. Each row in the response consists of the *Δ* vector for the PB in question. As all individuals in the training data are PB, all elements of **Y** ∈ {0, 1}, i.e., dummy variables for the breed.

In principle, all predictor variables, i.e., SNPs, are included but assigned different weights, defined by the loading matrix **P** (*p* × *m*). The score matrix **T** = **XP**, a *n* × *m* matrix, defines the relevant subspace of **X**, where *m* is the number of relevant components.

We used *m* = *q* − 1 where the reasoning is that two breeds will be well separated on one axis/component, three breeds by two axis, or in general *q* breeds by *m* = *q* − 1 axis/components, where each PB should represent one node point, and one node point only, in the *m*-dimensional space spanned by the scores.

A frequently used method for dimension reduction is principal component analysis (PCA) (Pearson, 1901). In contradiction to PLS, PCA is an unsupervised method, not taking breed information into account when constructing the scores. For comparison of the two methods, the first four scores from PCA and PLS are compared and evaluated against the prerequisite that each PB should represent one node point, and one node point only, in the space spanned by the scores.

PLSR predictions might yield results whose elements are larger than 1 or smaller than 0. These elements were truncated to 1 − 10^−10^ and 10^−10^, respectively, for the evaluation of Kullback–Leibler divergence.

The R-package “pls” (Liland et al., 2021) was used for fitting PLS and PCA models. The response (**Y** as described previously), predictors (**X** as described previously), and number of components (*q* = 4 for TrainP+ and *q* = 3 for TrainP−) were the data/parameters used as inputs for fitting the PLS and PCA models.

#### 2.4.3 Partial least squares with quadratic discriminant analysis

The principles of classification and discriminant analysis (DA) are given in Hastie et al. (2009). The goal is to find a posterior probability for different classes (CBs):

$$P\left(K|x\right)=\frac{f_{k}\left(x\right)\pi_{k}}{\sum_{l=1}^{n_{\mathrm{comb}}+1}f_{l}\left(x\right)\pi_{l}},$$ (1)

where *f*_*k*_(**x**) is the class-conditional density of **x** (observed SNPs), assuming that the SNP sample is from an individual of class *K*, and *π*_*k*_ is the prior probability of class *K*.

For PLS-DA, **x** is replaced with **t**, i.e., the PLS score vector. Furthermore, a common assumption is to assume that *f*_*k*_(**t**) is (multivariate) normally distributed with different mean parameters (*μ*_*k*_). When variance is assumed to be constant among classes, the method is known as linear discriminant analysis (LDA), which is notably applied on PLS scores (PLS-LDA) (Boulesteix, 2004). In the present study, we assume different variance parameters *Σ*_*k*_ for each class, which is known as quadratic discriminant analysis (QDA) (Hastie et al., 2009). Initially, this assumption is applied to the scores of the PB individuals, i.e., the PLS scores (*t*_*j*_) for PB individuals are assumed to be multivariate normal:

$$t_{j}∼N_{m}\left(\mu_{j},\Sigma_{j}\right),j=1,\ldots ,q,$$ (2)

where *q* is the number of PBs.

For the problem in the present study, only data from PB animals are used for training, and we lack observations, i.e., score vectors, for all CB classes. In order to implement CB classes, we need to find the class-conditional densities (*f*_*k*_(**x**)) for CB classes, without having the realization of score vectors for these classes. In addition, we included a class “unknown,” i.e., an unknown breed combination, leading to *n*_*comb*_ + 1 possible classes. The inclusion of the “unknown” class is possible for PLS-QDA, due to its Bayesian nature, where possible CB combinations are defined *a priori*, in contradiction to RF and PLSR.

A natural assumption is to assume that the scores of CB animals are distributed as linear combinations in accordance with the proportion of inherited DNA from the PB F0 generation. Let *θ*, a vector of length *q*, represent the proportion of DNA material in an F2 individual inherited from grandparents of different F0 PB individuals. Then, 0 ≤ *θ*_*j*_ ≤ 1, *j* = 1, …, *q*, and $\sum_{j}^{q}\theta_{j}=1$ . Under the assumption that *θ* is known for a CB individual, it is natural to model $t=\sum_{j}^{q}\theta_{j}t^{j}$ , where **t** ^*j*^ is the score associated with PB class *j*. Using standard proprieties of the normal distribution, we have

$$t|\theta ∼N_{m}\left(\overset{q}{\underset{j=1}{\sum }}\theta_{j}\mu_{j},\overset{q}{\underset{j=1}{\sum }}\theta_{j}^{2}\Sigma_{j}\right).$$ (3)

The proportion of DNA inherited from each grandparent is not exactly equal to a quarter. Thus, *θ* might be viewed as a random variable with *E*(*θ*) = *Δ* and defined variance *V*(*θ*). By applying the law of total expectation (Adam’s law) and variance (Eve’s law), we find that

$$\begin{array}{cc} E\left(t\right) & =E_{\theta }\left[\overset{q}{\underset{j=1}{\sum }}\theta_{j}\mu_{j}\right]=\overset{q}{\underset{j=1}{\sum }}\delta_{j}\mu_{j}=\left[\mu_{1}\ldots \mu_{q}\right]\Delta =\mu^{+}\Delta ,\\ V\left(t\right) & =E_{\theta }\left[\overset{q}{\underset{j=1}{\sum }}\theta_{j}^{2}\Sigma_{j}\right]+V_{\theta }\left[\mu^{+}\theta \right]=\overset{q}{\underset{j=1}{\sum }}\left(V\left(\theta_{jj}\right)+\delta_{j}^{2}\right)\Sigma_{j}+\mu^{+}V\left(\theta \right){\mu^{+}}^{t}, \end{array}$$ (4)

where *V* (*θ*_*jj*_) is the *j*th diagonal element of *V* (*θ*_*l*_) and the columns of the *m* × *q* matrix *μ* ^+^ are given by the *q* expectation vectors (*μ*_*j*_) for PBs. We will assume, even if it is an approximation, that the marginal distribution for **t** is multivariate normal with expectation and variance as given in Eq. 4, i.e.,

$$t∼N_{m}\left(\mu^{+}\Delta ,\overset{q}{\underset{j=1}{\sum }}\left(V\left(\theta_{jj}\right)+\delta_{j}^{2}\right)\Sigma_{j}+\mu^{+}V\left(\theta \right){\mu^{+}}^{t}\right).$$ (5)

Ordinary least squares (OLS) estimates are used for *μ*_*j*_ and *Σ*_*j*_, i.e., sample means and sample covariance for the PB PLS score vectors from the training datasets. In addition, *V*(*θ*) needs to be estimated. This was done by assuming that *θ* is Dirichlet distributed with the concentration parameter *α*_0_ *Δ*; consequently, *E*(*θ*) = *Δ*, and $V\left(\theta \right)=\frac{1}{\alpha_{0}+1}\left(d\left(\Delta \right)-\Delta \Delta^{t}\right)$ , where *d*(*Δ*) is the diagonal matrix with *Δ* on the main diagonal. Then, the only unknown parameter is *α*_0_, which was estimated by the method of moments on simulated data. A total of 1,000 simulations of *θ*, applying *Δ* as a vector of quarters, was conducted by IBD simulation (Vigeland, 2021); see Section 2.2 for details. The diagonal elements of the simulated variance have expected values $3{\left(16\alpha_{0}+16\right)}^{-1}$ leading to $\hat{\alpha_{0}}=3{\left(16\hat{\mathrm{Var}\theta }\right)}^{-1}-1$ , where $\hat{\mathrm{Var}\theta }$ is the mean diagonal element of the empirical variance based on simulated data, which is affected by several factors, including the genetic map (Tortereau et al., 2012).

We assumed that the class-conditional density of **t** for the unknown breed (funknown′′(t)) was uniform over the *q* − 1-dimensional space spanned by the range of PB score vectors.

We use two different prior distributions, i.e., *π*_*k*_ in Eq. 1, a “flat prior” for different breed-combinations, i.e., $\pi_{1}=\ldots =\pi_{n_{\mathrm{comb}}+1}=\frac{1}{n_{\mathrm{comb}}+1}$ and an informative prior where *π*_*k*_ is set equal to the proportion of pig litters of crossbreed *k* among all pig litters in Norway in 2021 (Langaker et al., 2021). The PLS-QDA soft prediction is given by $\hat{\Delta }=\sum_{k=1}^{n_{\mathrm{comb}}}\Delta_{k}P\left(K|x\right)$ , where *P*(*K*|**x**) is the posterior probability for class *K*, and *Δ*_*k*_ is the associated breed proportion vector; see Eq. 1. For the class unknown, we used *Δ* = 0_*q*_.

PLS-QDA models were fitted and evaluated in RStudio (Posit team, 2023) with custom functions, where the package “mvtnorm” (Genz et al., 2023) was used extensively. Codes are available at GitHub (Gangsei et al., 2023), a repository which also contains codes for replicating results, tables, and figures in the present study. For a more extensive exploration of the classification results presented in this article, an R-Shiny app has been made available (Gangsei, 2023).

## 3 Results

### 3.1 Comparing PCA and PLS

**FIGURE 2:**
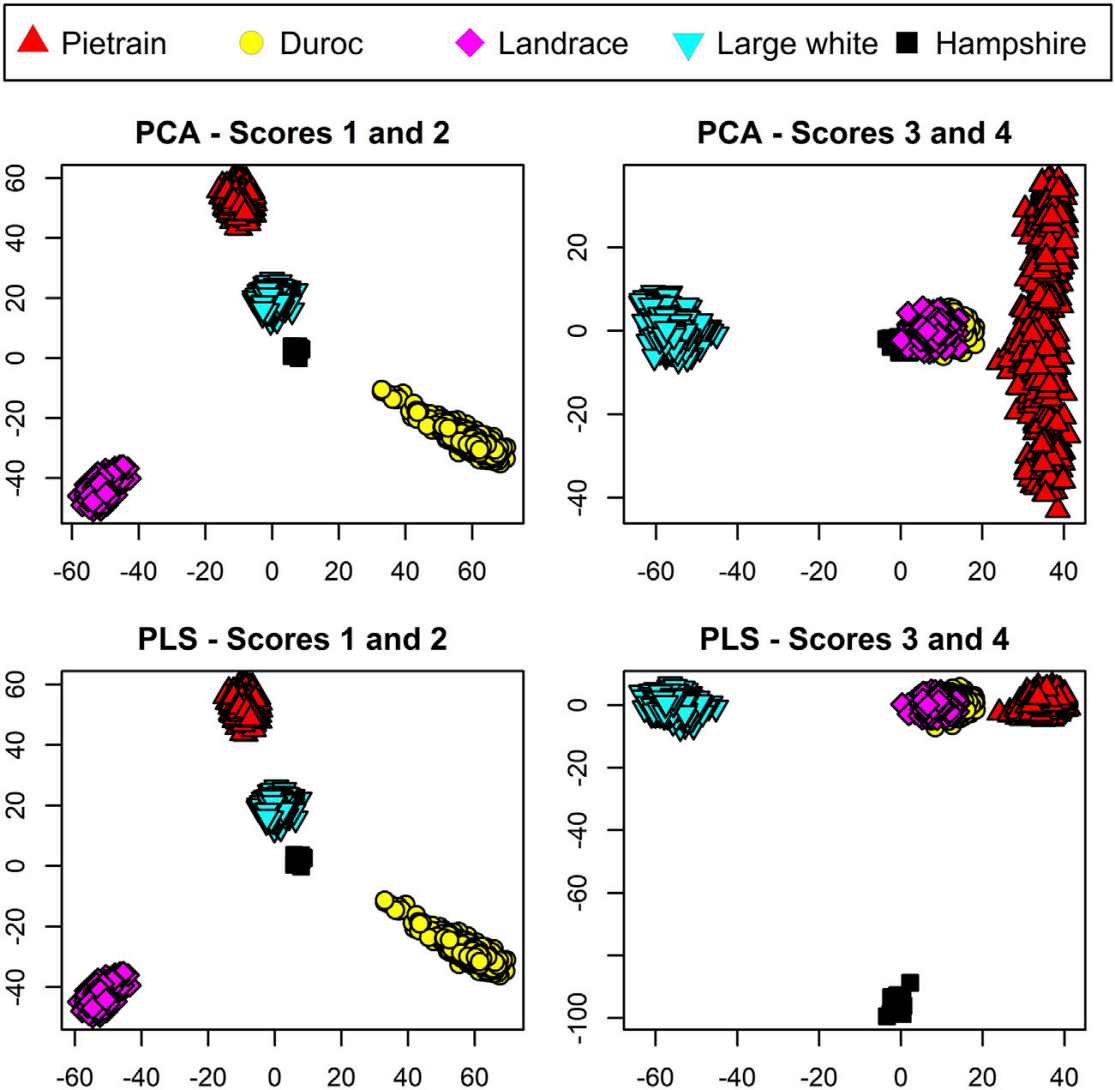
Score plots for pure breed animals based on the four primary scores using principal component analysis (PCA) displayed in the upper panels and partial least squares (PLS) displayed in the lower panels. Pietrain pigs are represented in red, Duroc in yellow, Landrace in magenta, Large White in cyan, and Hampshire in black.

For visualization of the data, both PCA and PLS were conducted on the TrainP+ data. The results are shown in Figure 2. For both PCA and PLS, first and second components both split the three breeds Duroc, Landrace, and Pietrain in a similar way. Component 3 manages to separate Large White from the other breed classes for both PCA and PLS approaches. The difference lies in the last, small breed (*n* = 14) Hampshire. The fourth component from the PCA mainly spans within variation of Pietrain and Hampshire, i.e., the breed with a small sample size cluster from the other breeds but does not represent its own node point in the four-dimensional space spanned out by the first four PCA components. In contradiction, the fourth component from PLS manages to distinguish this pig breed with its own node point even with a small sample size. As CBs are regarded fuzzy sets, each PB should represent node points in the *m* = *q* − 1 dimensional space spanned by the scores in order to prevent equal center points (*μ*) for different breed combinations. PCA fails to incorporate this prerequisite for the unbalanced dataset, and hence, PCA is not included in further analysis. The total variance explained (*R* ^2^) by the first four components in TrainP+ was 34.4% and 34.9% of the *X*-matrix for PLS and PCA, respectively. For both PLS and PCA, *R* ^2^ > 99.1% for all PBs except Hampshire, which had *R* ^2^ = 90.6% and 5.0% for PLS and PCA, respectively. For TrainP−, the cumulative *R* ^2^ values for three components were 32.1% and 32.5% (for *X* with PLS and PCA), >99.2% for all PBs except Hampshire with *R* ^2^ = 91.1% and 5.9% for PLS and PCA respectively.

### 3.2 Classification of simulated data

#### 3.2.1 Comparing methods

Table 1 displays an overview of the classification results based on the simulated data, for soft and hard classification with both the Kullback–Leibler divergence and the square loss. In general, PLS-QDA performed best as it managed to correctly classify 6,901 and 6,892 (KL-dist and Sq-loss, respectively), or 99% average, out of the total of 7,000 individuals when trained with Pietrain (TrainP+) and tested for all combinations (TestP+ and TestP−). Similar results for ADMIXTURE and PLSR are 6,744 and 6,826 (97% average), and 6,768 and 6,840 (97% average), respectively. RF, by far, performed worst as it only correctly classified 458 and 2073 (17% average) individuals.

**TABLE 1:** Evaluation of prediction errors for soft predictions as mean ± standard deviations and hard predictions given as numbers of correct classifications as the total number and % (*n* = 3,500 for TestP+ and TestP−) based on Kullback–Leibler divergences and quadratic errors, crossed over the two training (TrainP+ and TrainP−) sets and test sets (TestP+ and TestP−).

Combination	Method	RF	ADMIXTURE	PLSR	PLSQDA
TrainP+ TestP+	KL-dist (soft)	0.31 ± 0.2	0.072 ± 0.088	0.038 ± 0.038	0.0015 ± 0.013
	KL-dist (hard)	300 (8.6%)	3,326 (95%)	3,438 (98%)	3,458 (99%)
	Sq-loss (soft)	0.061 ± 0.033	0.01 ± 0.01	0.0084 ± 0.0093	0.0016 ± 0.012
	Sq-loss (hard)	1,208 (35%)	3,404 (97%)	3,424 (98%)	3,455 (99%)
TrainP+ TestP−	KL-dist (soft)	0.46 ± 0.24	0.053 ± 0.063	0.064 ± 0.08	0.0024 ± 0.016
	KL-dist (hard)	158 (4.5%)	3,418 (98%)	3,330 (95%)	3,443 (98%)
	Sq-loss (soft)	0.075 ± 0.041	0.0073 ± 0.0092	0.0084 ± 0.0096	0.002 ± 0.015
	Sq-loss (hard)	865 (25%)	3,422 (98%)	3,416 (98%)	3,437 (98%)
TrainP−TestP+	KL-dist (soft)	12 ± 16	12 ± 16	12 ± 16	9.6 ± 16
	KL-dist (hard)	0 (0%)	0 (0%)	0 (0%)	0 (0%)
	Sq-loss (soft)	0.29 ± 0.26	0.28 ± 0.29	0.26 ± 0.27	0.37 ± 0.24
	Sq-loss (hard)	0 (0%)	0 (0%)	0 (0%)	0 (0%)
TrainP−TestP−	KL-dist (soft)	0.3 ± 0.18	0.048 ± 0.065	0.056 ± 0.081	0.0024 ± 0.017
	KL-dist (hard)	595 (17%)	3,400 (97%)	3,208 (92%)	3,445 (98%)
	Sq-loss (soft)	0.05 ± 0.035	0.0077 ± 0.0098	0.0085 ± 0.0098	0.0025 ± 0.021
	Sq-loss (hard)	1,627 (46%)	3,408 (97%)	3,406 (97%)	3,434 (98%)

**FIGURE 3:**
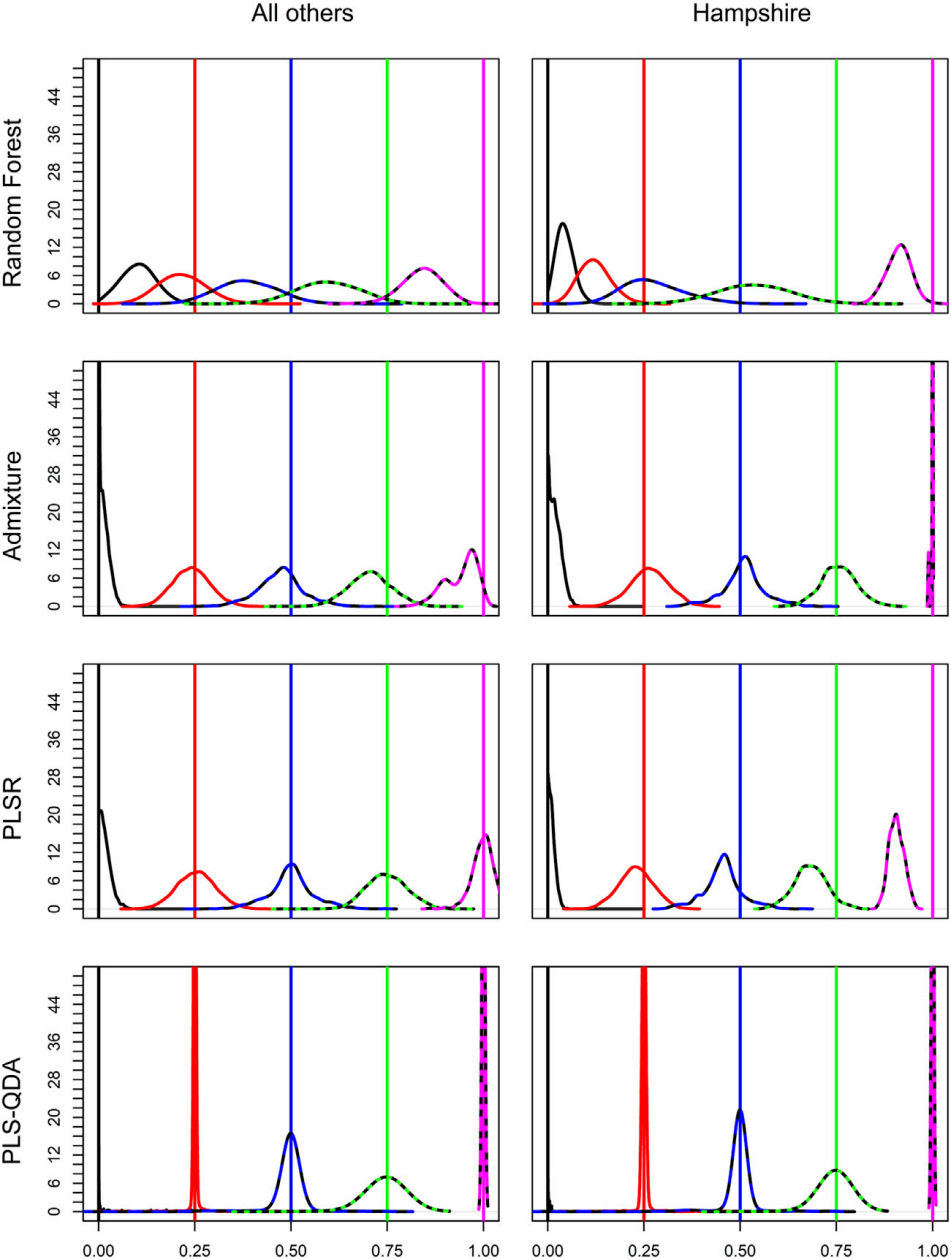
Densities for elements in the soft classifications $\left(\hat{\Delta }\right)$ . The left column represents results obtained from breeds Duroc, Landrace, Pietrain, and Large White, and the right column represents results from Hampshire. The rows represent the four different methods, i.e., RF, ADMIXTURE, PLSR, and PLS-QDA. The curves show empirical densities for $\hat{\delta_{j}}$ for *δ*_*j*_ = 0/4, 1/4, *…*, 4/4, with colors black, red, blue, green, and magenta, respectively. Densities are based on results from models trained on the training set TrainP + and applied to both simulated test sets, i.e., TestP+ and TestP−. Vertical lines represent the true proportions, i.e., *δ*_*j*_.

Soft classification results for different methods are visualized in Figure 3. The figure shows results based on all breed combinations except Hampshire in the first column. All methods have best precision for PBs, i.e., PB proportion for breed *j* (*δ*_*j*_ = 1), or when PB is not present at all, i.e., *δ*_*j*_ = 0. For *δ*_*j*_, at 0.25, 0.5, and 0.75, the classification precision decreases with increasing *δ*_*j*_ for PLS-QDA, indicating that PB proportions of 0.75 are most poorly classified with PLS-QDA. Hampshire results are of particular interest as only 14 individuals were present in the training data compared to 1,000 individuals for the four other breeds. Even if RF performs poorest overall, it is more noticeable for Hampshire than the other breeds as the Hampshire proportions are heavily underestimated by RF. To some extent, this is also the case for PLSR, while ADMIXTURE and PLS-QDA seem to yield unbiased estimates also for Hampshire proportions.

#### 3.2.2 Effect of omitting breed from training data

All methods performed well when trained and tested on their respective “alike” datasets, as well as when trained with Pietrain and tested without, as shown in the second row in Table 1.

Contrary to prior expectations, there do not appear to be large differences in the classification precision for TestP− depending on whether Pietrain is included in the training data (TrainP+) or not (TrainP−). When evaluated as the proportion of correct classified individuals, the results are marginally better with TrainP+ compared to TrainP−, in particular, for ADMIXTURE and PLSR. However, the average Kullback–Leibler divergence and square loss are, in general, marginally smaller with TrainP− compared to TrainP+. Consequently, including Pietrain in the training data does not seem to impair the model’s classification ability, even for CBs without Pietrain.

#### 3.2.3 Pietrain regarded as an unknown breed

To get an understanding of how inclusion of unknown breeds, i.e., breeds not present in the training data, in CB combinations affects the classification results for PLS-QDA, results for the combination TrainP− TestP+ were evaluated and are presented in Table 2. For this combination, Pietrain might be regarded as an “unknown breed” and, ideally, all individuals in TestP+ should be classified as “unknown” for the model based on TrainP+. In Table 2, “P” is still the abbreviation for Pietrain, but the combinations are generalized, i.e., “XXXX” = “DDDD,” “HHHH,” “LLLL,” “WWWW” (PB), “XXXY” = “DDDH,” …, “WWWL,” i.e., three grandparents of the same breed, etc. Most of the CB blends with Pietrain are recognized and classified as an “unknown” breed. PB Pietrain (“PPPP”), almost PB Pietrain (“PPPX”), and the CB of one Pietrain together with three of the four other classes (“PXYW”) are more likely to be classified as a mix of all the other four PB combinations (“XYLK”) with 100%, 46%, and 32% classified as “XYLK,” respectively. A likely explanation is that the center point for “DHLW” is close to origo, with a large associated generalized variance.

**TABLE 2:** Proportions (in %) of the predicted breed combination, i.e., maximum posteriori probabilities with PLS-QDA, for the model trained without Pietrain (TrainP−) vs. true breed combinations for the simulated test set with Pietrain (TestP+). Breed combinations are generalized, i.e., “XXXX” = {*“DDDD*,*” “HHHH*,*” “LLLL*,*” “PPPP*,*” “WWWW”*} (PB), “XXXY” = {*“DDDH”*, …, *“ZZZL”*}, i.e., three grandparents of the same breed, etc.

		Predicted combination (%)						
		Unknown	XXXX	XXXY	XXYL	XXYY	XYLK	n
True combinations	PPPP	0	0	0	0	0	100	100
	PPPX	54	0	0	0	0	46	400
	PPXX	100	0	0	0	0	0	400
	PPXY	80	0	0	0	0	20	600
	PXXX	66	28	6	0	0	0	400
	PXXY	68	0	17	8	6	0	1,200
	PXYL	41	0	0	27	0	32	400

### 3.3 General PLS-QDA results

The Bayesian method PLS-QDA, which might be regarded as a refinement of PLSR, performs best and also has more flexibility in its modeling, e.g., it can easily be implemented with an unknown breed combination. The support for classifying to “unknown” can be adjusted by changing the associated prior probability *π*_*Unknown*_. Changing *π*_*Unknown*_ has some similarities to changing the significance level for hypothesis testing. If the support for an unknown breed is lowered, i.e., *π*_*Unknown*_ is decreased, fewer individuals will be classified as “unknown breed,” i.e., the probability of classification to a “real CB class” is increased, with the disadvantage that the probability of incorrect classification is increased. The analogy to hypothesis testing is that the higher significance level increases the probability of rejecting the null hypothesis but also increases the risk of doing a type I error. Consequently, if it is of huge importance to avoid incorrect classifications, *π*_*Unknown*_ should be increased and vice versa.

Another feature which is unique for PLS-QDA is the possibility to use an informative prior. The effect of the informative prior on classification results for the real data, TestR, with PLS-QDA, trained on TrainP+, is shown in Table 3. The effect of the informative prior is conspicuous and as expected *a priori*. The number of individuals classified to the two dominating CB classes i.e., “DDLW” and “HHLW,” i.e., typically F1 commercial (“LW”) maternal line and Duroc (“DD”) or Hampshire (“HH”) paternal lines, increases, in particular, at the expense of the number classified as “unknowns.” PLS-QDA with an informative prior and ADMIXTURE yields close to similar results for the real data, with one exception, the “unknown group,” which is natural since classifying to “unknown” is not a feature in ADMIXTURE.

**TABLE 3:** Predicted breed combinations for the test set containing real data based on models trained on all data (TrainP+). Predictions as posterior maximums applying the informative and flat prior to the PLS-QDA method and hard predictions based on the square loss for ADMIXTURE. The last columns show differences in the total number classified to different CBs for PLS-QDA with and without informative priors and ADMIXTURE. The group “other combinations” contains sums for breed combinations with fewer than 10 predictions for any of the methods.

	Number of breed combinations			Difference between methods		
	Flat	Informative	ADMIXTURE	Inf. vs. Adm	Adm. vs. Flat	Inf. vs. Flat
DDLW	497	532	531	1	34	35
HHLW	169	192	193	−1	24	23
Unknown	118	70	0	70	−118	−48
LLWW	41	44	46	−2	5	3
DDLL	43	43	53	−10	10	0
LLLL	38	39	41	−2	3	1
LLLW	23	24	22	2	−1	1
HHLL	17	16	36	−20	19	−1
DLPW	16	0	0	0	−16	−16
LWWW	10	0	11	−11	1	−10
HLLW	0	0	14	−14	14	0
Other combinations	41	53	66	−13	25	12

Estimates for *μ*_*j*_ and *Σ*_*j*_ in Eq. 2 are PB-specific means and covariances based on the PLS scores. The PLS scores for PBs used for these calculations are shown in the two lower panels in Figure 2. *μ*_*j*_ and *Σ*_*j*_ for CBs are linear combinations of *μ*_*j*_’s and *Σ*_*j*_’s for PBs, as shown in Eq. 5.

A crucial success factor for PLS-QDA is the incorporation of additional variance in CB covariance due to the stochastic nature of the proportion of DNA inherited from grandparents. Table 4 shows average matrix determinants at the log scale for the theoretical covariance matrices, as given in Eqs 4, 5. The size of the determinant of the covariance matrix is referred to as the generalized variance by Wilks (1932) and might be viewed as a scalar describing the size of the covariance matrix in question. The generalized variance increases with an increasing number of PBs in the CB combination. For comparison, the log scale determinants of empirical variances from predicted scores, i.e., **t** ^*sim*^ = **X** ^*sim*^ **P**, where **X** ^*sim*^ is the matrix of simulated SNPs, are shown in the same table. The results show determinants of the same size for both training sets, except for PBs where the variance based on empirical scores is smaller.

**TABLE 4:** Mean ± standard deviation for covariance matrix determinants using a logarithmic scale, crossed over generalized breed combinations i.e., “XXXX” = {*“DDDD*,*” “HHHH*,*” “LLLL*,*” “PPPP*,*” “WWWW”*} (PB), “XXXY” = {*“DDDH*,*”* …, *“ZZZL”*}, i.e., three grandparents of the same breed, etc., and if Pietrain is included in the analysis or not. The column “mixed normals” is based on Σ in the likelihood function, and the column “Simulated data” is based on empiric covariances from simulated data.

	Generalized breed combination	Mixed normals	Simulated data
P+	XXXX (*n* = 5)	5 ± 1.3	2.4 ± 0.63
	XXXY (*n* = 20)	5.8 ± 0.72	5.4 ± 0.48
	XXYL (*n* = 30)	7.1 ± 0.38	7.8 ± 0.45
	XXYY (*n* = 10)	5.5 ± 0.45	6 ± 0.36
	XYLK (*n* = 5)	9.1 ± 0.25	9.7 ± 0.26
P−	XXXX (*n* = 4)	4 ± 1.2	2 ± 0.51
	XXXY (*n* = 12)	5.3 ± 0.62	4.8 ± 0.48
	XXYL (*n* = 12)	7 ± 0.3	7.3 ± 0.19
	XXYY (*n* = 6)	5.2 ± 0.45	5.5 ± 0.39
	XYLK (*n* = 1)	9.3 ± NA	8.7 ± NA

The behavior of classification results, center points (*μ*), and associated covariance (*Σ*) for CBs of different complexities and different settings for the informative prior and support for unknown breed (*π*_*Unknown*_) can be explored in the R-Shiny app (Gangsei, 2023).

## 4 Discussion

The overall aim of this study was to evaluate crossbreed classification of commercial finisher pigs based on genomic data from a 50-K (Illumina) SNP chip, with the four different methods, namely, RF, ADMIXTURE, PLSR, and PLS-QDA. The novelty was to implement PLS-QDA as an alternative method with several beneficial features to analyze the genomic SNP data.

PLS was used as an alternative dimension reduction method to PCA due to its additional features. Subsequent theoretical deductions led to the extended method, PLS-QDA. For comparison, two methods not built on dimension reduction were also executed: the model-based ADMIXTURE, which is a well-functioning software application for ancestry classification, and the well-known classification method RF. The classification results, for all methods on the two simulated test sets with and without an additional breed, show that PLS-QDA had the highest accuracy and that PLSR and ADMIXTURE are both methods that meet prior expectations to classification accuracy.

It was observed, naturally, that all methods have best precision for the classification of individual elements, *δ*_*j*_ in *Δ*, when the element is associated with a PB, i.e., *δ*_*j*_ = 1, or when the PB is not present at all, i.e., *δ*_*j*_ = 0. For PLS-QDA, it was observed that the classification precision for *δ*_*j*_ was decreased at 0.25, 0.5, and 0.75, indicating that PB proportions at 0.75 are most poorly classified with PLS-QDA. A similar pattern is not evident for ADMIXTURE; however, the results for PB proportions at 0.75 are approximately equal for PLS-QDA and ADMIXTURE.

PLS-QDA has some advantages compared to ADMIXTURE and PLSR. First, it performs best when tested on the simulated data, even if this is by small margins, but more important is its ability to incorporate an unknown breed combination. The results presented in Table 2 show that the PLS-QDA method is capable of classifying CBs with Pietrain grandparents as “unknowns” to a large degree. The exception is PB Pietrain, almost PB Pietrain (“PPPX”), and the CB of one Pietrain together with three of the four other classes (“PXYW”), where many individuals were classified as a mixture of the four other PBs. Consequently, classification results with four different PBs should be interpreted with caution as it may be an unknown purebred not seen in the training set. Some misclassifications of CBs with other levels of Pietrain grandparents also occur; however, in general, the method performs reasonably well for these combinations, in particular for individuals with 50% Pietrain grandparents.

Another advantage PLS-QDA has in comparison to the other methods is the possibility to use different prior distributions for the CB populations. These priors might change in time and space, for instance, in other target populations, e.g., countries with other dominating breed combinations. By assigning high prior weight to the unknown breed group, more individuals will be classified as unknowns at the expense of the known CBs. As argued in the results, the interpretation of the prior weight for an unknown breed has similarities to the interpretation of the significance level in hypothesis tests, i.e., a higher significance level/lower prior for unknown breed not only leads to increased strength for classifying a known CB/reject the null hypothesis, but also an increased possibility of misclassification/type I errors. This is a desirable feature enabled by the Bayesian nature of QDA and, to the best of our knowledge, a novelty in classification of crossbreed pigs.

A main result is that, at least for unbalanced data, PLS is preferable over PCA as PLS fulfills the prerequisite of assigning one node point in the *q* − 1-dimensional space to each PB, which is, thus, a necessity for classification purposes and, in addition, is a considerable advantage for visualization. This is illustrated in Figure 2 where PLS assigns one PB to each node point in the *q* − 1-dimensional space defined by the scores. This is a prerequisite for the PLS and PLS-QDA methods as CBs are considered blends in a space where the PBs represent the extremes. For purely practical purposes, this might have been solved by having a more balanced dataset, i.e., the same number of Hampshires as for the other breeds. However, the insight has significance since new breeds might fairly easily be added to the model without the need for observations from a large number of individuals when PLS is used.

It should be recognized that PLS, at least for genomic data such as the data used in the present study, fulfills the need for dimension reduction, with better results than PCA in the sense of more dense and dispersed clusters of PBs for the first *m* PCA/PLS components. As a key finding of the present study, we highlight that PLS might be used as a complementary method for dimension reduction of SNP data under the assumption that a “supervising” feature in the present study “breed,” is available.

An important feature of a general method is the stability when exposed to new or unknown breeds that are not included in the PB F0 generations. The results show that both PLS-QDA and PLSR, as well as ADMIXTURE, are flexible in the sense that new PBs might be added to the training data without substantial loss with respect to classification accuracy, even if some of the included PBs are not present in the target population. As strongly anticipated, classification performance for breeds not included in training data was poor. However, the results showed marginal deterioration when the method was trained with the new breed, TrainP+, and classified without, TestP−. Hence, the disadvantage of training on a variety of PBs is small even if the possible crossbreed combinations are well known *a priori*. Consequently, for all methods, other PBs might be included in the training data, with small or even negligible loss of classification precision in populations where one or more of PBs is not present. The possibility of adding new PBs to the model without the need for a large sample size for PBs in question is a highly desirable feature for PLSR, PLS-QDA, and ADMIXTURE.

RF performed, by far, the poorest for classification of the simulated data, in particular for CBs, including Hampshire. This is in line with prior assumptions as Hampshire was hugely underrepresented in the training data. RF is built on tree prediction, where a considerable number of trees collectively favor the class with the highest probability. With few observations in the training datasets, Hampshire will most likely not be included in the training of all tree models, and therefore, RF will give a skewed result and suffer when presented with a small class in the test data. RF performance depends on the tuning of hyper parameters. In the present study, hyper parameters were tuned to values giving OOB errors at 0 in the training set and at the same time yielded small computational cost. It is not unlikely that RF performance could be improved more by extensive tuning of the hyper parameters based on the model’s performance on the simulated test data. However, as models for all methods were fitted based on training data only, the same principle should be applied to RF. The results show that for the present study, RF is a sub-optimal classification method, most likely due to the unbalanced data structure. This could also be adjusted in favor of RF if operated with more balanced data, but as argued previously, it is beneficial with methods that perform well on unbalanced data and balanced for generalization purposes. The three other methods, at least PLS-QDA and ADMIXTURE, seem to be robust against the unbalanced training dataset and without extensive hyper parameter tuning.

Two different measures, namely, square loss and Kullback–Leibler divergence, were both used for two purposes: comparing the accuracy of soft predictions between methods, and transformation of soft predictions to hard predictions for RF, ADMIXTURE, and PLSR. Formally, the transformations from soft to hard predictions based on the square loss and Kullback–Leibler divergence are just discriminant classification performed on the soft predictions, $\hat{\Delta }$ , with a flat prior for CBs. The square loss corresponds to LDA, and Kullback–Leibler is an alternative discriminant function. The results from both methods are quite similar but differ marginally. We view it as a strength that the evaluation of the results seems to be affected to a negligible degree by the choice of distance measurement.

The purely practical applications for the models included in the present study are limited to breed and breed combinations for the five PBs included, i.e., Duroc, Hampshire, Landrace, Large White, and Pietrain. However, through the results and principles, we show that both ADMIXTURE and PLSR/PLS-QDA are methods where other PBs might be fairly easily included, even when PB data for new breeds are scarce, which are key findings in the study. Another limitation to the study is the unbalanced training set, containing only 14 pigs of the breed Hampshire. This has been seen as an opportunity to evaluate the methods in a more realistic setting than a balanced dataset would provide. Therefore, it has been kept this way intentionally instead of pruning the data by, for instance, taking out Hampshire as PB.

In the present study, breed combinations, not breed permutations, were used as classifying units. Variation in the percentage of DNA material inherited from PB animals is affected by different breed permutations under the same breed combination. By only considering combinations, potential information associated with different permutations might be lost. For example, consider the combination *“LLWW”* consisting of the six permutations *“LLWW”*, *“LWLW”*, *“LWWL”*, *“WLLW”*, *“WLWL”*, and *“WWLL”*. When calculating mean (*μ*_*j*_) and covariance (*Σ*_*j*_) for CBs (see Eq. 5), the assumption is that the proportion of DNA inherited from grandparents, i.e., *θ*, was Dirichlet distribution with the concentration parameter *α*_0_ *Δ*, leading to $V\left(\theta \right)=\frac{1}{\alpha_{0}+1}\left(d\left(\Delta \right)-\Delta \Delta^{t}\right)$ , where *Δ* represents the breed combinations in the F0 generation. This assumption seems reasonable for all permutations; however, for the two permutations *“LLWW”* (F1 commercial maternal line) and *“WWLL”*, both with two PB individuals in the F1 generation, we know that the proportion of DNA inherited from the two PBs in question is 50% exactly, which is not the case for the other four permutations. Consequently, for “*LLWW*” and “*WWLL*”, θ = Δ = [0 0 ½ ½ 0]^*t*^ (i.e., zero variance for *θ*). For the four other permutations, it is natural to assume non-zero variance for *θ*. The consequence, referring to Eq. 5, is that covariance, *Σ*_*j*_, associated with permutations *“LLWW”* and *“WWLL”* should be smaller than the other permutations, as all elements including *V*(*θ*) in Eq. 5 should be excluded for these permutations. This information might be possible to utilize in order to, at least to some extent, distinguish different breed permutations under the same breed combination. However, the strength of classifying different permutations is likely to be low as the means, i.e., *μ*_*j*_ in Eq. 5 are unaffected by *V*(*θ*). The effect of permutation clustering within combinations is easy to observe for real data classified as breed combination *“HHLZ”* in the 3D Shiny app (Gangsei, 2023). These individuals cluster inside their associated limiting spheres. From prior information, it is overwhelmingly likely that the only permutation existing within this combination is the crossing of the Hampshire paternal and TN70 maternal lines, which also highlights that permutations might be identified by an informative prior.

The software program used for the simulation of data in the study was developed with a primary area of application for the human genome, in particular kinship analyses and forensic genetics. Due to the genetic map provided by Tortereau et al. (2012), it was possible to apply the software application to the pig genome in a realistic manner. The genetic map is averaged over sex and four different breed combinations (“pedigrees”) containing PBs Large White, Meishan, Yorkshire, Berkshire, Duroc, and Landrace. The recombination rates varied between breed combinations and sexes (Tortereau et al., 2012). Consequently, the use of an average genetic map in the present study is an approximation. However, the effect of variations in the genetic map is assumed to be of minor importance as it will only have limited effects on the parameter *α*_0_ scaling the variance of the proportion of the inherited genomic material from the four grandparents (*θ*). Higher recombination rates would yield larger values for *α*_0_ and lower variance for *θ*. In the present study, *α*_0_ was kept constant at its estimated value at 73.58. A possible topic for future research is to evaluate the effects of changing this value and thereby the covariance matrices for CBs.

A challenge with the SNP data is that they only contain information regarding the two nucleobases that are present at each SNP but no information regarding whether the nucleobases originate from the paternal or maternal line. For homozygote SNPs, this data structure causes no problems. For heterozygote SNPs, the two nucleobases were randomly assigned to the maternal or paternal chromosomes of the F0 generation when assigning the nucleobases to a simulated IBD chromosome structure.

For the simulation study, breed permutations were drawn randomly within each breed combination. A topic for future studies might be to design simulations for different breed permutations and apply a classification model for permutations based on theoretically different variances, in order to classify permutations within the same combination. Such studies would, to the best of our understanding, be of more theoretical than practical interest.

The simulation study provides SNP simulations for CBs and behaves as a credible realization for SNP data in real CB individuals. This is supported, although not proved, by the fact that simulated data are distributed in accordance with the model, both regarding expected values and variances. Consequently, it is reasonable to assume that the evaluation of methods based on the simulated results, to a great extent, describes the real precision and reliability for different methods and breed combinations. To explore how PLS-QDA and ADMIXTURE behave when applied to a real example, the trained PLS-QDA and ADMIXTURE models were tested on real data, TestR. The distribution of CB classes was in accordance with prior knowledge, i.e., the dominating CBs were *“DDLW”* and *“HHLW”*, even when using the flat prior for PLS-QDA. The flat vs. informative prior results for the PLS-QDA method appear to be a textbook example of how an informative prior might be utilized in a Bayesian setting. The inclusion of the informative prior has a substantial effect by allocating more individuals to breed combinations known to be dominating *a priori*, at the expense of the “unknown” class and CBs known to be rare *a priori*. Still, the informative prior does not totally dominate the classification results. ADMIXTURE classifies closer to PLS-QDA with informative priors, which may indicate that the method is adequate in adjusting for actual populations. The prior information can neither be added nor changed. This result again advocates for ADMIXTURE as a reliable method for classification. It could be interesting to see how the two different methods, PLS-QDA with informative priors and ADMIXTURE, behave on real data from other real situations with other CB combinations.

Some of the real data are classified to CBs containing Pietrain, even if Pietrain should not be present in the Norwegian pig population. The CBs with Pietrain are *“DLPW”* and *“HLPW”*, i.e., four breed combinations. Inclusion of some genetics of Pietrain origin cannot be totally ruled out in Norway; however, for the last 15–20 years, the policy of breeding companies operating in Norway has been to avoid using Pietrain genetics. From the simulation results, we observed that PB individuals from different breeds were not part of the training set and were generally classified as four-breed combinations. Consequently, the four-breed classification results should be interpreted with care as they might, in fact, be PBs or close to PB individuals, from breeds not included in the training dataset. In Norway, at least the Mangalica breed is present and, in fact, a possible candidate for these classifications. A natural development of the work presented in this study would be to incorporate Mangalica as a new PB in the training data.

The results in this study can beneficially be used for generalization to other problems in several ways. The simulation tool showed an excellent generalization from humans to pigs and can be generalized to other breeds/populations/countries or to other species with genomic data available in the form presented in the current study. Prior knowledge of recombination rates, i.e., the study of Tortereau et al. (2012), was essential for the present study, both in the simulation and in order to estimate *V*(*θ*) and thereby *Σ*. If similar information regarding recombination rates is available, the methods described in the present study might be transferred to similar problems for other species, assuming that genomic data are available.

Another interesting topic, which falls outside the scope of this study, is to consider other responses than breeds. For instance, a feature such as color could be treated in a similar way, where some colors are viewed as references, i.e., the counterpart to PBs in the current study and other color combinations as blends, i.e., the counterpart to CBs.

ADMIXTURE and RF were tested as possible candidate methods. Other candidates could also have been included, for instance, different classification methods that deal better with unbalanced data. Although RF failed as a real candidate, ADMIXTURE performed well for both simulated data and real data. Thus, the result of this study confirms ADMIXTURE’s suitability as a standard software program for classifying genetic origins, not only for human ancestry. Kim et al. (2022) indicate how ADMIXTURE, in combination with PCA, behaves nicely and provides useful information for both classification and visualization in a pig population.

Partial least squares with linear discriminant analysis (PLS-DA) has recently been shown to perform well on other problems with similar SNP data (Miao et al., 2023). The derivation of PLS-QDA for CBs was initiated and conducted prior to the publication by Miao et al. (2023). However, PLS-QDA might be viewed as an elaboration of PLS-DA utilized in Miao et al. (2023) in the sense that i) PLS-QDA was applied to CB classification in contrast to PLS-DA used for PB classification only, and ii) the derivation of CB-specific covariance matrices is a prerequisite for QDA and novel to the present study. In particular, a research topic for further analysis could be to apply PLS-QDA and simulation of CBs to the data used in Miao et al. (2023) where the number of PBs was much higher than that in the present study (*n* = 91).

The main focus of the study was to evaluate the PLS-based methods, in particular to derive equations for the expected values *μ*’s and covariances used in the likelihood functions for CBs. Another important objective was to show that PLS extends and improves classification in a more robust way for unbalanced data and when faced with unknown breed combinations, which is a reality when working with real data from slaughterhouses.

## 5 Conclusion

In the present study, it has been shown that PLS-QDA, PLSR, and ADMIXTURE are well suited methods for the crossbreed classification of pigs based on genomic data from a 50-K (Illumina) SNP chip from purebred grandparents. ADMIXTURE is a well-proven method that is suited for ancestry classification tasks with genetic SNP data. It originates from kinship in humans but proved to work nicely and was easy to transfer to pigs. The method of the main focus in the present paper, PLS-QDA, has some advantages compared to the other methods. It has the highest classification accuracy, which supports the inclusion of an “unknown breed combination” class and an informative prior. Finally, it facilitates informative visualization in 3D format. Accurate CB classification has important applications, in particular, related to research and development topics in the pig industry, including breeding progress, carcass grading, meat yield, and quality. Another important contribution from the current study is to incorporate the stochasticity in the proportion of inherited DNA from ancestors as a feature utilized for PLS-QDA as an extension of PLS-DA.

## Data Availability

The original contributions presented in the study are included in the article, further inquiries can be directed to the corresponding author.
